# Hidden allies: how extracellular vesicles drive biofilm formation, stress adaptation, and host–immune interactions in human fungal pathogens

**DOI:** 10.1128/mbio.03045-23

**Published:** 2024-11-18

**Authors:** Philipp Brandt, Rima Singha, Iuliana V. Ene

**Affiliations:** 1 Institut Pasteur, Université Paris Cité, Fungal Heterogeneity Group, Paris, France; Instituto Carlos Chagas, Curitiba, Brazil

**Keywords:** extracellular vesicles, fungal pathogens, biofilms, drug resistance, immunity, stress response

## Abstract

Pathogenic fungi pose a significant threat to human health, especially given the rising incidence of invasive fungal infections and the emergence of drug-resistant strains. This requires the development of vaccines and the advancement of antifungal strategies. Recent studies have focused on the roles of fungal extracellular vesicles (EVs) in intercellular communication and host-pathogen interactions. EVs are nanosized, lipid membrane-bound particles that facilitate the transfer of proteins, lipids, and nucleic acids. Here, we review the multifaceted functions of EVs produced by different human fungal pathogens, highlighting their importance in the response of fungal cells to different environmental cues and their interactions with host immune cells. We summarize the current state of research on EVs and how leveraging this knowledge can lead to innovative approaches in vaccine development and antifungal treatment.

## EXTRACELLULAR VESICLES FROM HUMAN FUNGAL PATHOGENS

Fungal pathogens pose a significant risk to human health, causing approximately 6.5 million invasive infections and 3.8 million deaths yearly (1–3). The populations most vulnerable to systemic fungal infections include individuals with compromised immune systems, such as those with HIV/AIDS, patients undergoing cancer therapy, or those receiving immunosuppressive agents (4). In response to the growing threat of fungal infections to public health, the World Health Organization published its first fungal pathogen priority list including *Candida albicans*, *Cryptococcus neoformans*, *Candida auris*, and *Aspergillus fumigatus* in the critical priority group (5, 6). The high mortality rates of fungal infections, the lack of vaccines, and the limited diagnostics and antifungal therapies underscore the urgent need for a better understanding of fungal pathogenesis and comprehensive strategies to mitigate the impact of fungal infections on public health (1, 5, 7).

Recent studies on microbial pathogenesis have highlighted the growing importance of EVs in cell-to-cell communication and host–pathogen interactions (7–11). EVs are nanoscale bilayered lipid-bound organelles secreted by cells in the extracellular space. Initially envisioned as a mechanism for cells to excrete unwanted materials (12), our appreciation of the importance of EVs has grown significantly. Indeed, EVs are ubiquitous across kingdoms and participate in both normal physiology and pathological states. EVs transfer biomolecules, such as proteins, lipids, nucleic acids, and carbohydrates from their parent cells, playing crucial roles in inter-microbial communication, disease transmission, microbe–host interactions, and microbial adaptation to environmental stress (9, 11, 13). Owing to their small size and immunogenic properties, EVs are currently being explored as potential biomarkers for disease progression and vehicles for drug delivery (14).

## FUNGAL EVs ARE HETEROGENEOUS IN SIZE AND CARGO COMPOSITION

Multiple fungal species have been reported to produce EVs, including both pathogens and non-pathogens from the genera *Candida* (15–19)*, Cryptococcus* (20), *Histoplasma* (15), *Aspergillus* (21), *Fusarium* (22), *Sporothrix* (23), *Paracoccidioides* (24), *Malassezia* (25), *Pichia* (26), *Trichophyton* (27), *Talaromyces* (28), *Neurospora* (29), *Zymoseptoria* (30), and *Saccharomyces* (31). Unlike the EVs produced by other eukaryotic cells (32), fungal EVs have not been classified into distinct subpopulations (e.g., exosomes, microvesicles). However, their characterization has revealed high heterogeneity concerning size, morphology, cargo, and function. The size of fungal EVs ranges from 10 to 1,000 nm and differs among species and mode of growth (e.g., planktonic versus biofilm growth, yeast versus hyphal forms, liquid versus solid culture) (33–35). For example, *C. albicans* biofilm EVs range from 30 to 200 nm in diameter, whereas planktonic EVs have two populations with diameters of 30–200 and 200–1,000 nm (36). Another study highlighted that *C. albicans* EVs derived from yeast cells are significantly larger, ranging from 400 to 500 nm in diameter, with a smaller subset around 100 nm. In contrast, hyphal-derived EVs are predominantly smaller, with diameters ranging from 100 to 200 nm (37). Other *Candida* species, including *Candida tropicalis*, *Candida parapsilosis*, *Candida glabrata*, and *C. auris* produce biofilm EVs similar in size to those of *C. albicans* (33). The average size of *C. neoformans* EVs is less than 100 nm in diameter, but these vesicles are highly heterogeneous in shape, including spherical, tubular, flat, and multilayered EVs (35). In some species (e.g., *C. albicans* [35], *Saccharomyces cerevisiae* [35], *A. fumigatus* [21], *C. neoformans* [35]), subpopulations of EVs are coated with fibrillar structures, which are hypothesized to be composed of cell wall polysaccharides, such as mannoproteins, and therefore could mediate interactions with the host immune system (38, 39).

Concerning cargo composition, fungal EVs contain diverse lipids, proteins, nucleic acids, carbohydrates, pigments, and small molecules. Proteomic data sets from several species report the presence of proteins associated with metabolism, cell wall biogenesis, plasma membrane, and stress responses. Moreover, the EV cargo is modulated by the growth conditions and the environment to which the parent cells are subjected. For example, EVs from *C. neoformans* cells grown on a nutrient-deficient medium rather than those from cells grown on a rich medium contained higher levels of virulence factors including the capsular polysaccharide glucuronoxylomannan (GXM) and the enzymes laccase and urease (40). Similarly, the size of *H. capsulatum* EVs and their ergosterol and phospholipid content varied when the cells were grown in brain heart infusion (BHI), Ham’s, or RPMI media (41).

In *C. albicans*, the EV proteome contains several highly enriched proteins that could represent potential EV markers for this species, including proteins from the claudin-like Sur7 family (42). As many of these proteins are fungal-specific, they could be leveraged to track EVs and to study their biogenesis and cargo loading. Planktonic and biofilm *C. albicans* EVs differed significantly in their cargo, with 34% of the proteins being unique to the biofilm state. Biofilm EVs were rich in mannans and glucans, two polysaccharides that are also enriched in the biofilm extracellular matrix (36). Interestingly, the composition of biofilm vesicles closely resembled that of the biofilm matrix in terms of protein and polysaccharide content, suggesting that vesicles may be a significant source of matrix material (36). Hyphal extracellular vesicles in *C. albicans* contain a more diverse array of proteins relative to those derived from yeast cells. These include virulence factors, metabolic enzymes, transport proteins, and components of the endosomal sorting complexes required for transport (ESCRTs), which are involved in exosome biogenesis. In contrast, yeast-derived extracellular vesicles predominantly carry cell wall-associated proteins (37). Differences between different *Candida* species have also been noted. For example, EVs from *C. auris* cells showed increased amounts of lysophospholipids and lower levels of ergosterol and lipids involved in energy storage (e.g., di- and tri-acylglycerols) relative to *C. albicans* EVs (43). Their proteomic profile also differed, particularly for carbon metabolism, indicating that EVs have different functions in the metabolic adaptation of the two species (43). Moreover, several immunogenic proteins were identified in *C. auris* EVs, such as homologs of Adh1, Mp65, and Xog1 (43). The analysis of EV proteomes of *C. albicans*, *C. tropicalis*, *C. parapsilosis*, *C. glabrata,* and *C. auris* revealed that the protein cargo varies significantly among species and mirrors their phylogenetic relationships (33). The study also identified a group of 36 common proteins, which was enriched for factors involved in biofilm development, biofilm dispersion, extracellular matrix, and drug tolerance, including proteins, such as Bgl2, Xog1, Sun41, Cht3, Mp65, Tos1, and Zrt2 (33). Thus, while EVs have similar biological functions and a subset of shared proteins, the recruitment of machinery necessary for these processes can differ significantly among species.

In *Cryptococcus*, a core set of 17 proteins were common in *C. neoformans, Cryptococcus deneoformans,* and *Cryptococcus deuterogattii*, and these included factors involved in cell wall remodeling (such as chitin and glucan enzymes), amylase, oxidases, and membrane proteins (such as those from the tetraspanin SUR7/PalI family) (35). Analysis of *S. cerevisiae* EVs revealed that they are not enriched with proteins from the ESCRT, unlike mammalian EVs, but that they contain cell wall integrity proteins, including enzymes involved in the breakdown and restructuring of polysaccharides, such as glucan and chitin synthases (e.g., Fks1, Chs1, Chs3) (44). Similarly, mass spectrometry analyses of *H. capsulatum* vesicles revealed several phospholipids as the major lipid species and diverse proteins involved in glucan biosynthesis, endocytosis, heat shock, as well as lipid, protein, and carbon metabolism (41). Thus, the EV cargo of fungal pathogens holds a diverse repertoire of proteins and lipids, and many of these molecules contribute to biofilm formation, metabolic adaptation, stress responses, and fungal virulence.

## THE EV CARGO IS LOADED WITH CELL WALL REMODELING PROTEINS

**Fig 1:**
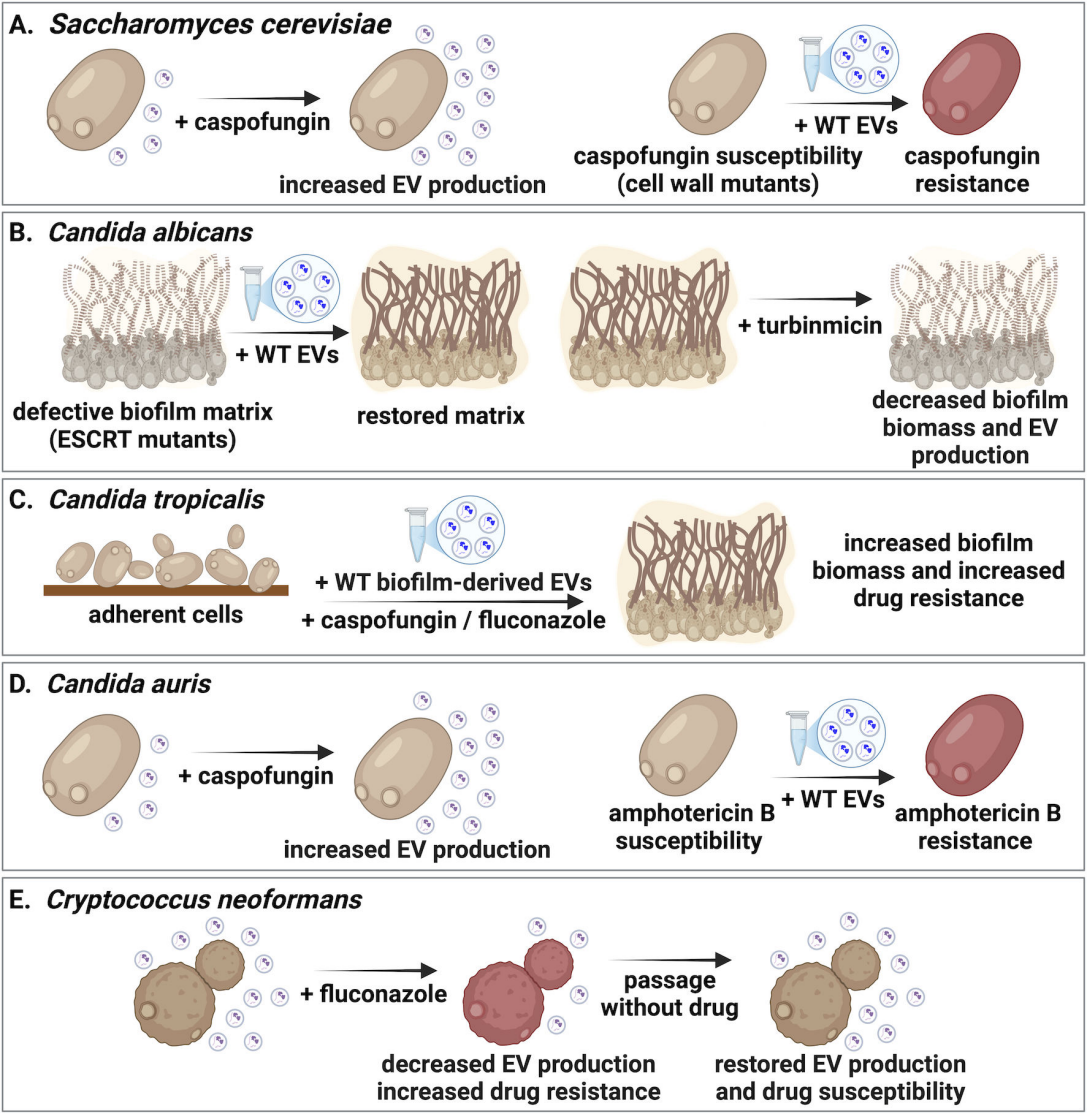
The role of fungal EVs in antifungal resistance. (**A and D**) In *S. cerevisiae* and *C. auris*, caspofungin treatment leads to increased EV production. Complementation assays using wild-type EVs can restore the caspofungin susceptibility of cell wall defective mutants in *S. cerevisiae* and increase resistance to amphotericin B in *C. auris*. (**B and C**) In *C. albicans* and *C. tropicalis*, EVs contribute to drug resistance, biofilm formation, and the production of the biofilm extracellular matrix. Inhibitors of the vesicular trafficking pathway, such as turbinmicin, decrease biofilm biomass and EV production in *C. albicans*. In *C. tropicalis*, complementation with biofilm-derived EVs increases drug resistance and biofilm biomass. (**E**) In *C. neoformans*, fluconazole exposure decreases EV yield. Serial passaging in the absence of antifungal pressure restores EV production and drug susceptibility. Illustration created with BioRender.

The ability to rapidly adapt to cell wall stress is essential for fungal survival in the host and in the presence of antifungal drugs (45). As the cell wall could act as a barrier for EV export, the weakening of the cell wall by deleting key cell wall biosynthetic enzymes was postulated to increase the secretion of vesicles. Proteomic analysis of *S. cerevisiae* EVs revealed the presence of 24 proteins involved in cell wall remodeling, including seven glucanases and three glucanosyl-transferases, as well as several cell wall degrading enzymes, supporting the idea that vesicle passage through the cell wall may involve breaking down structural components (31). Indeed, deleting a major cell wall chitin synthase encoded by *CHS3* and the 1,3-β-glucan synthase subunit encoded by *FKS1* resulted in increased EV release in *S. cerevisiae* (44). Similarly, treating yeast cells with caspofungin, which inhibits β-glucan synthesis, enhanced EV secretion (Fig. 1A) (44). Moreover, the addition of EVs from wild-type cells was able to protect caspofungin-sensitive *chs3*∆ cells from the detrimental effects of this antifungal (Fig. 1A) (44).

The cargo of *C. albicans* EVs is also enriched with proteins related to cell wall organization, including the enzymes Bgl2, Crh11, Pga4, Phr1, Phr2, chitin and glucan synthases such as Chs3 and Fks1, mannoproteins, and GPI-anchored cell wall proteins (42). Similarly, the core EV proteins identified in *Cryptococcus* species include cell wall processing enzymes, such as Gas1 (a 1,3-β-glucanosyltransferase), Amy1 (an α-amylase), Exg104 (a glucan 1,3-β-glucosidase), Hep2 (a putative heparinase), and Cda (a chitin deacetylase), suggesting that *Cryptococcus* EVs could also be involved in cell wall architecture (35).

Several cell wall remodeling proteins were found in the EV proteome of *Paracoccidioides brasiliensis*, including β-glucan and mannan enzymes, an α-1,3-glucan synthase involved in carbohydrate remodeling, as well as the signaling GTP-binding protein Rho1, involved in the cell wall integrity pathway (46). Similarly, *H. capsulatum* vesicles are packed with proteins involved in cell wall architecture, such as chitin, glucan, and mannan enzymes, and a protein kinase C receptor involved in cytoskeleton organization (15). Therefore, the EV proteome from several fungal pathogens is enriched with cell wall remodeling enzymes and signaling proteins that EVs can deliver to participate in cell wall repair. These reports also point to a conserved role for EVs in cell wall integrity across fungal species from different genera.

## EVs PLAY CENTRAL ROLES IN BIOFILM FORMATION AND DRUG RESISTANCE

Fungal EV biogenesis and cargo loading are enabled by the ESCRT-mediated multivesicular body (MVB) pathway (8, 47). In *C. albicans*, multiple (16 out of 17 tested) mutants lacking homologs of ESCRT subunits displayed reduced EV production, and several of these (7 out of 17 tested) showed reduced biofilm formation upon treatment with the antifungal drug fluconazole (36). Furthermore, the addition of wild-type EVs restored the extracellular matrix architecture and drug resistance of several ESCRT mutants, demonstrating that biofilm EVs contain functional protein cargo (48) (Fig. 1B).

Interestingly, the cargo of biofilm-derived EVs acts in multiple stages of biofilm formation, including adhesion and dispersion as wild-type EVs can compensate for mutant phenotypic defects and provide community coordination during biofilm development (48). EVs from other *Candida* species, such as *C. tropicalis*, *C. parapsilosis*, *C. glabrata,* and *C. auris* showed similar roles in biofilm formation, biofilm dispersal, and drug tolerance (33) (Fig. 1C). Cross-species vesicle addition of wild-type EVs restored the biofilm-forming abilities of biofilm-deficient strains in EV complementation assays, indicating that EVs also have roles in multispecies biofilms (33). In addition, turbinmicin, a drug that targets Sec14 and inhibits vesicular trafficking was tested for its impact on biofilm formation by *C. albicans*, *C. tropicalis*, *C. glabrata, C. auris*, and *A. fumigatus* (49, 50). Turbinmicin treatment reduced vesicle production in a dose-dependent manner and decreased biofilm biomass both *in vitro* and in a rat catheter model of *C. albicans* infection (49) (Fig. 1B). Drug combinations of fluconazole and turbinmicin exhibited strong synergy compared with either agent alone, leading to nearly complete biofilm disruption (50). This effect was likely due to extracellular matrix damage and enhanced fluconazole permeability (50). Thus, EVs contribute to biofilm matrix formation and consequently to drug resistance.

The role of EVs in biofilm formation has also been studied in *C. tropicalis*. The addition of *C. tropicalis* EVs at the initial stages (post-adhesion) of biofilm formation increased the biofilm thickness and metabolic activity upon fluconazole or caspofungin exposure, demonstrating that EVs can be protective against antifungal treatment (51) (Fig. 1C). The EVs from multidrug-resistant *C. auris* isolates have also been reported to contribute to drug resistance. A dose-dependent increase in amphotericin B resistance was observed upon the addition of EVs to this species (Fig. 1D) (34). This effect was specific to amphotericin B and was not observed for fluconazole, voriconazole, micafungin, or flucytosine (34). Moreover, supplementation with *C. albicans* EVs did not increase the amphotericin B resistance of *C. auris* (34). At the same time, the addition of *C. auris* EVs did not increase amphotericin B resistance in *C. albicans*, indicating a species-specific effect (34). In addition, caspofungin treatment led to an increase in both the size and quantity of released *C. auris* EVs, and altered their RNA and protein cargo, enriching them with factors involved in cell wall integrity and stress response processes (52). The mechanisms underlying these responses are still unclear, but these observations emphasize the multifaceted roles of EVs in drug resistance.

In *C. neoformans*, screening of a transcription factor deletion library identified four genes that altered both EV production and fluconazole resistance (53). Thus, strains lacking *HAP2, GAT5, LIV4,* or *BZP2* produced less than 1/10 of EVs relative to wild-type cells and showed altered sensitivity to fluconazole (53). Mutants of *HAP2*, *GAT5*, or *LIV4* showed decreased fluconazole susceptibility relative to the wild-type strain, while *BZP2* mutants showed increased susceptibility (53). The study found that EV production decreased ~2.4-fold when cells were grown in the presence of subinhibitory concentrations of fluconazole (Fig. 1E), without altering the expression of genes associated with fluconazole resistance. This indicated that the increased EV production represents an alternative mechanism of drug resistance (53). The study also showed that fluconazole-resistant isolates exhibited reduced EV production relative to susceptible isolates from the same clonal lineage (Fig. 1E) (53). Sequential passages of these isolates in the absence of antifungal led to the loss of drug resistance and restored EV production (Fig. 1E), demonstrating a direct correlation between EV production and antifungal resistance. It is evident that EVs from various fungal pathogens play a role in biofilm formation and antifungal drug responses. However, the mechanisms driving these processes and their potential conservation across fungal species remain to be explored.

## FUNGAL EVs AS STRESS MESSENGERS

Several reports indicate that the fungal EV cargo contains proteins involved in stress responses and mediate cell communication during biofilm formation, hinting that EVs act as stress messengers in pathogenic fungi. In *P. brasiliensis*, the genes *HACA* and *IRE1* belonging to the unfolded protein response (UPR) pathway were upregulated in cells incubated with EVs derived from tunicamycin-treated cells relative to EVs from untreated cells (54). The UPR pathway is activated to reestablish endoplasmic reticulum homeostasis by increasing folding capacity and controlling the disposal of misfolded proteins. Similarly, EVs derived from UV-irradiated *A. fumigatus* cells induced a distinct stress response in fresh *A. fumigatus* cultures. This response, marked by the upregulation of the *mpkC* stress response gene and the *akuA* DNA repair gene, differed significantly from that triggered by control EVs (54).

Heat shock proteins are released by cells upon thermal stress and act to maintain the cellular proteome, thereby protecting the cell from the effects of the heat stress. In *Talaromyces marneffei*, heat shock protein (HSP) and peroxidase have been identified in the EV cargo (28). Similarly, HSP (C0NVB8) and HSP60-like protein (C0P0B3) were found in EVs of *H. capsulatum* (55). In *Cryptococcus*, the EV cargo is enriched with antioxidants, heat shock proteins such as Hsp70, Hsp90, and a superoxide dismutase (56). Interestingly, *S. cerevisiae* uses EVs to transmit factors that enhance survival under heat stress. Disrupting exosome biogenesis by targeting the ESCRT machinery blocked this effect and proteomics analysis revealed that heat shock protein 70 (HSP70) family proteins, particularly Ssa2, are crucial for transferring thermotolerance (57). These findings highlight the conserved role of fungal EVs in heat stress response and proteostasis.

During candidiasis, oxidative stress in the host is likely to influence *C. albicans* EV release and cargo. To understand how oxidative stress impacts the EV cargo, menadione was added to *C. albicans* cultures, and metabolomics analysis of EVs identified lipid molecules involved in glycerophospholipid and sphingolipid pathways. These pathways were hypothesized to help *C. albicans* adapt to oxidative conditions during infection (58).

Overall, fungal EVs are likely to play crucial roles in stress response and cell communication, particularly during pathogenic interactions and biofilm formation. The presence of stress-related proteins, such as heat shock proteins and antioxidants, within the EV cargo across several fungal species underscores their potential conserved function in maintaining cellular homeostasis under stress conditions, such as heat or oxidative stress. These findings suggest that EVs act as messengers, enabling fungi to adapt to hostile environments by modulating stress response pathways. These studies also point to potential targets for therapeutic interventions, as manipulating EV biogenesis or cargo could disrupt fungal survival mechanisms during infection.

## FUNGAL EVs STIMULATE IMMUNE CELLS, INDUCE PRO-INFLAMMATORY RESPONSES, AND ENHANCE PHAGOCYTOSIS

**Fig 2:**
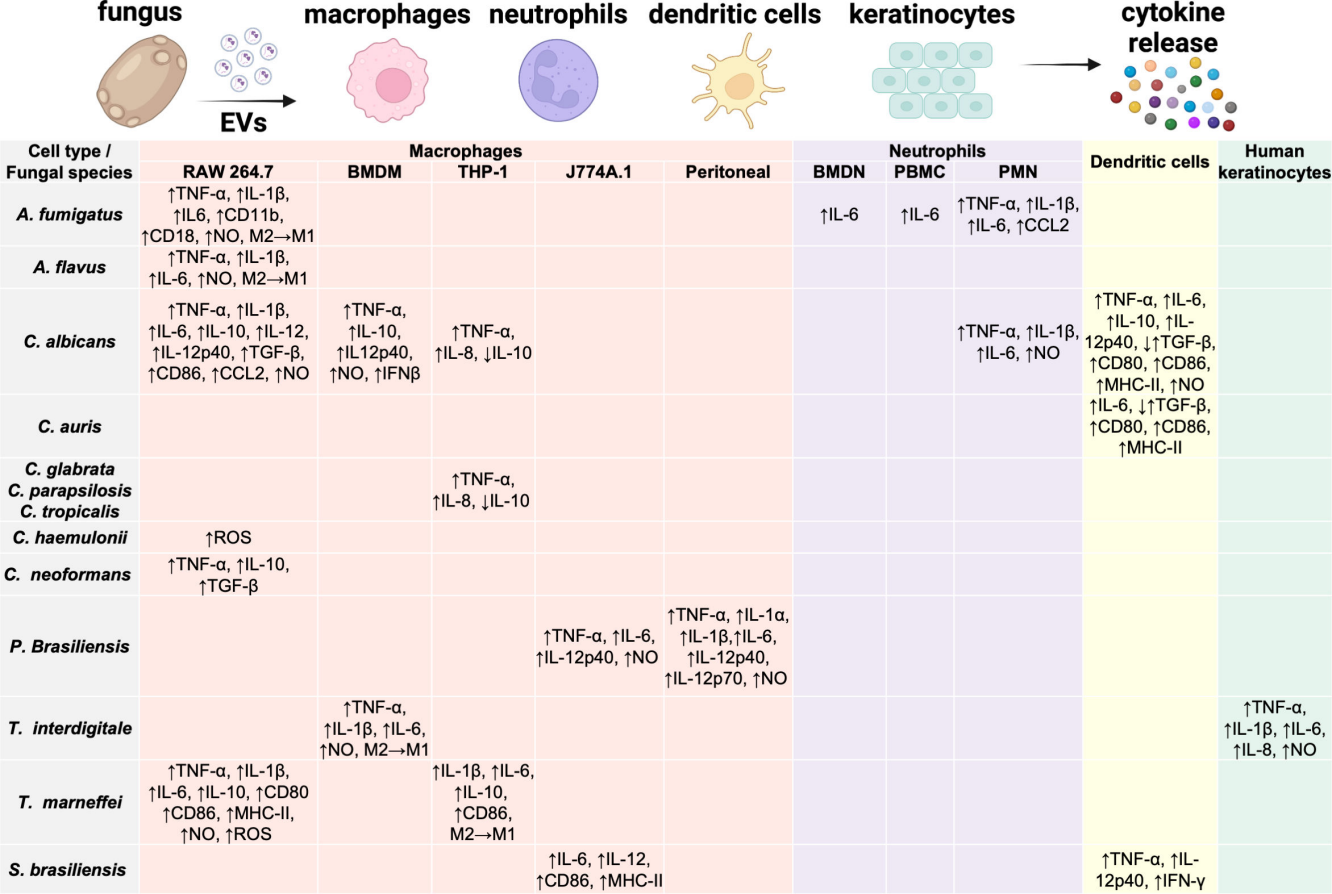
Fungal EVs modulate and interact with the host immune system. The table shows changes in cytokine expression, nitric oxide secretion, or phenotype transitions when specific immune cell types are stimulated with EVs isolated from different pathogenic fungi. Arrows indicate increases or decreases relative to no EV treatment. Empty spaces indicate that the respective immune responses have not been evaluated for EVs produced by those respective species. BMDM = bone marrow derived macrophages; BMDN = bone marrow derived neutrophils; PBMC = peripheral blood mononuclear cells; PMN = polymorphonuclear neutrophils. Illustration created with BioRender.

Fungal EVs exert significant effects on the host immune system, with the interaction varying depending on the fungal species, EV quantity, and type of immune cell involved. Across human fungal pathogens, EVs consistently stimulate immune cells, particularly macrophages, leading to the production of cytokines and immune mediators. Macrophages exposed to fungal EVs showed increased secretion of the tumor necrosis factor alpha (TNF-α) and nitric oxide. Besides this common response, the expression level of other cytokines appears dependent on the fungal species from which the EVs are derived (Fig. 2).

RAW 264.7 macrophages stimulated with either *A. fumigatus* or *Aspergillus flavus* EVs enhanced the expression of TNF-α, IL-1β, IL-6, and nitric oxide and induced macrophage polarization towards the M1 phenotype (59–62). The expression of the adhesion molecules CD11b and CD18 was increased in RAW 264.7 cells but not in AMJ2-C11 cells stimulated with *A. fumigatus* EVs and thus dependent on the macrophage cell type (61). The immune response of neutrophils to *A. fumigatus* EVs was also dependent on the neutrophil cell type. While bone marrow-derived neutrophils (BMDN) and neutrophils from healthy human donors enhanced the expression of IL-6 and did not induce the formation of neutrophil extracellular traps (NETs) (59, 61), polymorphonuclear neutrophils (PMNs) from mouse peritoneum increased the expression of TNF-α, IL-1β, IL-6, and CCL-2 after stimulation with *A. fumigatus* EVs (62). Furthermore, pre-treatment with *A. fumigatus*-derived EVs prior to infection with conidia enhanced the phagocytosis and clearance of conidia by both macrophages and neutrophils (59, 62). Similarly, pre-treatment with *A. flavus* EVs enhanced macrophage phagocytosis rates and increased the cytotoxicity against fungal conidia (60).

Various *Candida* species produce EVs that interact with and modulate the host immune system. High concentrations of *C. haemulonii* EVs increased reactive oxygen species (ROS) production in RAW 264.7 macrophages after internalization (63). However, *C. haemulonii var. vulnera* and its EVs did not display cytotoxic effects on RAW 264.7 macrophages. EVs produced by *C. glabrata*, *C. parapsilosis*, and *C. tropicalis* biofilms showed a common response by increasing TNF-α and IL-8 expression and decreasing IL-10 secretion after internalization by human THP-1 differentiated macrophage-like cells (51). *C. albicans* EVs were internalized by both macrophages (RAW 264.7, bone marrow-derived macrophages [BMDM]) and dendritic cells and induced nitric oxide production (17). For this species as well, the immune response was dependent on the type of immune cell stimulated—RAW 264.7 macrophages produced high levels of TNF-α, IL-1β, IL-6, IL-12, CCL2 and moderate levels of TGF-β and IL-10, whereas BMDMs showed high expression of IL-12p40, IL-10, TNF-α, and slightly elevated TGF-β (17, 64). In contrast, *C. albicans* yeast EVs from different genetic backgrounds increased the secretion of TNF-α and IL-8 while decreasing IL-10 in THP-1 macrophage-like cells (65). *C. albicans* EVs isolated from yeast cells did not induce cytotoxicity after internalization by THP-1 macrophages, while EVs from hyphal cells showed increased cytotoxicity and secretion of TNF-α (37). Thus, immune responses elicited by EVs depend on both fungal isolate and fungal morphology. Pre-treatment of RAW 264.7 macrophages with *C. albicans* or *C. auris* EVs prior to infection with yeast cells had no impact on phagocytosis, but enhanced macrophage killing of *C. albicans* cells but not that of *C. auris* cells (43). In contrast, another study showed that both the phagocytosis and killing ability of RAW264.7 macrophages increased when they were treated with *C. albicans* EVs prior to infection with yeast cells (64).

A combination of *C. albicans* yeast cells and EVs showed a synergistic effect by enhancing the cytotoxicity to RAW 264.7 macrophages, oral keratinocytes, squamous carcinoma cells (TR146), and gingival epithelial cells compared with infection with yeast cells or EVs alone (66). *C. albicans* EVs also stimulated the adaptive immune response by increasing CD86 expression (in macrophages and dendritic cells) and the major histocompatibility complex class II (MHC-II) in dendritic cells (17). Like macrophages, dendritic cells treated with EVs showed a significant increase in IL-12, IL-10, TGF-β, and TNF-α production (17). This was further demonstrated in another study: dendritic cells infected with either *C. albicans* or *C. auris*-derived EVs showed enhanced expression of MHC-II, CD80, and CD86 (43). In addition, EVs from both fungal species increased IL-6 and decreased TGF-β expression in dendritic cells (43). IL-6 production was significantly decreased in bone marrow-derived dendritic cells (BMDCs) lacking the Toll-like receptor 4 (TLR4) when these cells were treated with *C. albicans* EVs (39). THP-1 macrophage-like cells treated with *C. albicans* EVs also showed reduced IL-6 production when TLR4 was inhibited, indicating that EVs are primarily recognized by this receptor (39). *C. albicans* EVs can also modulate neutrophil function. Pre-treatment with *C. albicans* EVs enhanced the phagocytosis and killing of yeast cells by PMNs (64). In addition, *C. albicans* EVs enhanced the expression of TNF-α, IL-1β, IL-6, inducible nitric oxide synthase (iNOS), and the production of nitric oxide in a dose-dependent manner (64). Finally, a recent study showed that biofilm-associated *C. albicans* DNA found in EVs can activate the cGAS-STING pathway, leading to IFN-β production and the induction of interferon-stimulated genes (10).

Although *C. neoformans* EVs were the first EVs described in fungi (20), their interaction with immune cells has rarely been studied. *C. neoformans* EVs were internalized by RAW 264.7 macrophages which resulted in increased levels of TNF-α, TGF-β, and IL-10 and a dose-dependent increase of nitric oxide (67).

Treatment with *H. capsulatum* EVs prior to infection with yeast cells inhibited phagocytosis and intracellular killing of both BMDM and THP-1 macrophages (55).

EVs derived from the dimorphic fungus *P. brasiliensis* induced the production of nitric oxide and the pro-inflammatory mediators TNF-α, IL-6, and IL-12p40 during phagocytosis by J774A.1 or peritoneal macrophages, whereas the expression of IL-1α, IL-1β, and IL-12p70 was exclusively induced in peritoneal macrophages (68). *P. brasiliensis* EVs also induced switching from M2 to the M1 phenotype of peritoneal macrophages (68). In addition, macrophages treated with EVs prior to infection with *P. brasiliensis* showed increased killing (68). EVs from a virulent *P. brasiliensis* strain (isolated from granulomatous lesions) induced TLR4 and Dectin-1 expression in BMDMs and BMDCs and promoted an enhanced Th1/Th17 response in dendritic cells (69).

*Trichophyton interdigitale* is the first dermatophyte described to produce EVs (27). *T. interdigitale* EVs induced the production of proinflammatory cytokines TNF-α, IL-1β, IL-6, and nitric oxide in BMDMs when phagocytosed (27). Furthermore, the increased expression of iNOS indicates a polarization towards the M1 phenotype. Human keratinocytes infected with *T. interdigitale* EVs also induced the expression of TNF-α, IL-1β, IL-6, IL-8, and nitric oxide (27).

Recently, Yang et al. discovered and characterized EVs produced by the dimorphic species *T. marneffei*, formerly known as *Penicillium marneffei* (28). Internalization of *T. marneffei* EVs by RAW 264.7 macrophages resulted in increased expression of ROS, nitric oxide, TNF-α, IL-1β, IL-6, IL-10, and the co-stimulatory molecules CD80, CD86, and MHC-II (28). THP-1 macrophages infected with *T. marneffei* EVs also showed increased IL-1β, IL-6, IL-10, and CD86, and polarization to the M1 phenotype (70). In addition, pre-treatment of macrophages with *T. marneffei* EVs prior to conidia infection enhanced phagocytosis and fungicidal activity relative to non-treated macrophages (70).

Macrophages and dendritic cells infected with EVs from the emerging fungal pathogen *Sporothrix brasiliensis* did not show altered phagocytosis or cytokine production (23, 71). However, a co-culture of EVs and *S. brasiliensis* yeast cells significantly increased the expression of IL-6, IL-12, MHC-II, and CD86 in J774A.1 macrophages relative to macrophages infected with cells alone (71). Furthermore, the fungicidal activity was increased, whereas the phagocytosis rate was decreased in macrophages infected with a co-culture of EVs and yeasts (71). BMDCs infected with a co-culture of *S. brasiliensis* EVs and yeast cells showed increased phagocytosis and fungal burdens compared with dendritic cells infected with cells alone (23). In addition, the expression of TNF-α, IL-12p40, and IFN-γ was increased in dendritic cells infected with a co-culture of EVs and yeast cells (23). Thus, both phagocytosis and cytokine expression are dependent on the type of immune cell stimulated with EVs.

These studies indicate that EVs from human pathogenic fungi can stimulate immune responses, particularly in macrophages, leading to the production of cytokines and immune mediators. EVs appear to enhance phagocytosis and clearance of fungal cells, induce specific immune phenotypes, and activate both innate and adaptive immunity. The effects of EVs vary depending on the fungal species, fungal isolate, cell morphology, and immune cell type (Fig. 2). However, it remains unclear whether the *in vitro* ability of EVs to activate pro-inflammatory responses can be replicated *in vivo* during fungal infections. Confirming their role *in vivo* can position EVs as promising candidates for the development of antifungal vaccines.

## THE POTENTIAL OF FUNGAL EVs FOR IMMUNIZATION AGAINST FUNGAL INFECTIONS

**Fig 3:**
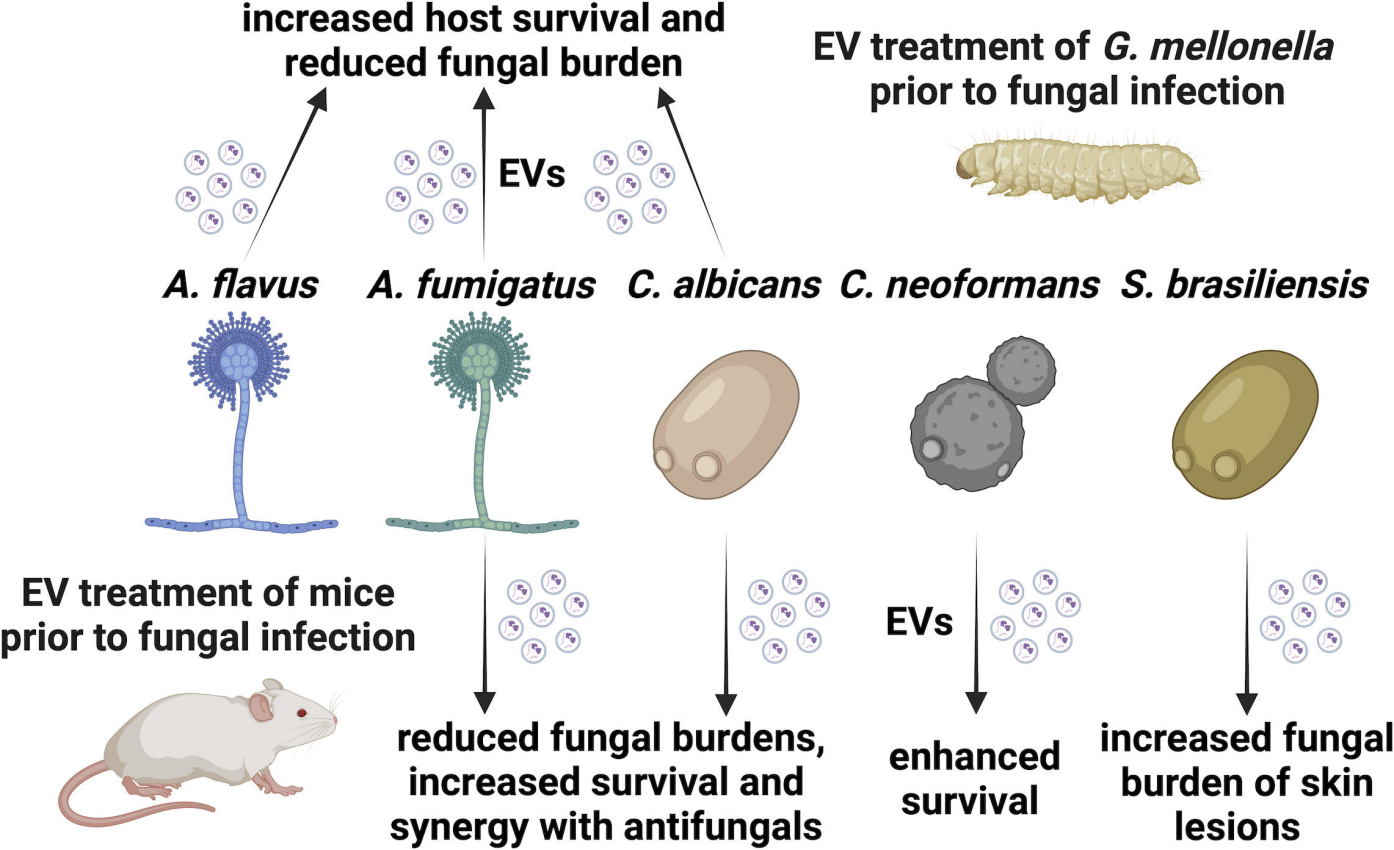
Animal immunization with fungal EVs protects against subsequent fungal infection. *G. mellonella* larvae treated with EVs derived from *A. fumigatus*, *A. flavus*, or *C. albicans* prior to fungal infection showed increased survival rates and reduced fungal burdens. A protective effect of fungal EV treatment prior to fungal infection was also observed in murine studies. Immunization with EVs derived from *A. fumigatus* and *C. albicans* increased fungal clearance, reduced fungal burdens in multiple organs, and showed synergistic effects with antifungals. While pre-treatment of mice with *C. neoformans* EVs enhanced survival in a cryptococcosis infection model, immunization with *S. brasiliensis* EVs increased the fungal load of skin lesions caused by this fungus. Illustration created with BioRender.

Despite multiple efforts, vaccines are still lacking as a strategy to prevent fungal disease. Given their immune-stimulating capacities, EVs were envisioned as a way to boost host defenses against a potential fungal infection. Thus, fungal EVs from different species have been tested for their vaccine potential by using *Galleria mellonella* and mouse models of infection (Fig. 3).

Pre-treatment of *G. mellonella* with either *A. flavus* or *A. fumigatus* EVs reduced fungal burdens and enhanced the survival of the larvae when infected with fungal conidia (60, 61). Similarly, pre-treatment with *C. albicans* EVs significantly enhanced *G. mellonella* survival and reduced fungal burdens during *C. albicans* infection (17). Interestingly, treatment of *C. albicans* yeast cells with its own EVs decreased fungal virulence in *G. mellonella*. While EV-treated *C. albicans* yeast cells led to 90%–95% of larval survival within less than a week, untreated yeast cells caused death in 80% of the larvae (72).

EV production also impacts the virulence of *C. gattii*. Deletion of the sugar transporters Past1 and Past2 resulted in a hypovirulent phenotype in a *G. mellonella* infection that was associated with reduced EV production and an altered EV cargo. Virulence was restored when mutant cells were co-injected with EVs produced by the wild-type strain (73). *C. neoformans* EVs derived from cells lacking *SGL1* (encoding for sterylglucosidase) that are enriched in sterylglucosides and glucuronoxylomannan (GXM), the major capsular polysaccharide of the fungus, enhanced the survival of *G. mellonella* upon *C. neoformans* infection (74). Another study showed that the vesicular peptide isoleucine–proline–isoleucine enhanced the survival of *G. mellonella* infected with lethal doses of either *C. gattii* or *C. neoformans* cells (75). Overall, these studies demonstrate that fungal EVs have protective effects in the *G. mellonella* infection model by reducing fungal burdens and enhancing host survival, especially when administered prior to infection. Additionally, mutants that affect the EV production or EV cargo can impact host survival, suggesting that EVs could be leveraged toward the development of new antifungal strategies.

In addition to *G. mellonella* larvae, the protective effect of EVs was investigated in various mouse infection models (Fig. 3). Mouse immunization with *A. fumigatus* EVs stimulated the production of antibodies (IgG), enhanced the phagocytosis and fungal clearance in the lungs, and reduced lung tissue damage of mice infected with *A. fumigatus* conidia (76). Furthermore, a combination of immunization with *A. fumigatus* EVs and amphotericin B treatment showed synergistic effects during pulmonary aspergillosis infection (76). EVs derived from *A. fumigatus* were neither toxic nor pathogenic for mouse cornea but enhanced the secretory IgA expression in mouse tear fluid (62). In addition, immunization with EVs reduced the fungal load and severity of *A. fumigatus* keratitis in infected mouse cornea which was associated with a reduction of the inflammatory factors IL-1β and CCL-2 (62).

Similarly, a subconjunctival injection of *C. albicans* EVs showed low toxicity to mouse cornea, and pre-treatment with *C. albicans* EVs reduced the severity of *C. albicans* keratitis in mice (64). *C. albicans*-derived EVs also showed a protective effect against murine candidiasis in immunosuppressed mice by reducing fungal burdens in the liver, spleen, and kidney and enhancing the survival of mice (39, 77). Vaccination of mice with *C. albicans* EVs was associated with increased TNF-α, TGF-β, IFN-γ, IL-4, IL-10, and IL-12p70 in the spleen of mice (77), thereby contributing to fungal clearance. The protective effect of *C. albicans* EVs in a mouse model of systemic candidiasis was lost in mice lacking TLR4, indicating that EV vaccination is TLR4-dependent (39).

The potential of *C. neoformans* EVs as a vaccine was further demonstrated in a murine model of cryptococcosis. Mice immunized with EVs from either the wild-type strain or the acapsular *cap59*∆ mutant showed increased survival compared with non-immunized mice upon *C. neoformans* infection (35). Notably, mice immunized with the acapsular mutant EVs survived longer than those immunized with wild-type EVs, indicating the involvement of cell wall components in EV immune recognition. In contrast, mice infected simultaneously with *C. neoformans* EVs and yeast cells showed increased traversal of the blood–brain barrier by *C. neoformans* in an *in vitro* model of brain microvascular endothelial cells (78). In addition, the fungal burdens were also increased in the brains of mice intravenously injected with a combination of *C. neoformans* EVs and cells (78). The outcome of the infection therefore depends on whether the mice are treated with EVs before infection with fungal cells or whether they are co-infected with EVs and cells.

Surprisingly, the fungal load of skin lesions in an *S. brasiliensis* subcutaneous mouse infection model was increased when mice were pre-treated with *S. brasiliensis* EVs (23). Thus, pre-treatment of EVs can also exacerbate fungal infections. Treatment of *H. capsulatum* with monoclonal antibodies (mAbs) that bind to HSP60 altered the size and cargo of EVs and significantly increased the survival of infected mice by reducing fungal burdens and organ tissue damage (79). mAbs also impacted the interaction between EVs and the host immune system. For example, macrophages treated with *H. capsulatum* EVs prior to infection with yeast cells showed reduced intracellular killing. This effect was potentiated when macrophages were treated with EVs derived from cells opsonized with a non-protective mAb (MAb 7B6) (79, 80). Thus, antibodies that modulate fungal EVs represent promising tools for the treatment of fungal infections.

Taken together, EVs from various fungal species show significant promise as innovative strategies for combating fungal infections. Immunization with fungal EVs can enhance survival, reduce fungal burden and tissue damage, stimulate immune responses, and synergize with antifungal treatments and monoclonal antibodies. The overall evidence supports the potential of fungal EVs for developing effective vaccines against fungal pathogens.

## UNLOCKING THE THERAPEUTIC CAPACITY OF FUNGAL EVs

The importance of EVs in fungal infections is only beginning to be appreciated (8). EVs act as mediators of cellular communication, influencing interactions between fungi and host cells, altering stress resistance, facilitating the transmission of virulence factors, and trafficking resources within fungal communities. Studying fungal EVs presents several key technical and biological challenges. A major hurdle is the isolation and purification of EVs, as fungal cells produce significant cell wall debris during growth, complicating the ability to obtain high-purity EV samples. Traditional methods like ultracentrifugation, while effective in mammalian systems, need to be combined with gradient purification to isolate clean EV populations. Additionally, standardizing protocols for EV characterization is challenging because fungal EVs vary in size, shape, and density depending on species and growth conditions, which complicates techniques like nanoparticle tracking analysis and electron microscopy. Moreover, the low EV yields from clinical or environmental samples limit comprehensive cargo profiling through proteomics or RNA sequencing. Finally, the absence of specific markers for fungal EVs makes it hard to distinguish them from other extracellular particles. Overcoming these challenges is critical for understanding fungal EV roles in pathogenesis and for developing potential therapeutic applications. Innovative imaging techniques and nanotechnologies are set to enhance our ability to visualize EVs in real-time and enable tracking their interactions with host cells. Future research on EVs will expand our understanding of fungal pathogenesis, interspecies communication, and host–pathogen interactions.

The study of fungal EVs as potential biomarkers for early diagnosis and as targets for innovative antifungal therapies is an exciting frontier. Owing to their small size and complex biomolecular properties, EVs represent an attractive tool for gene transfer, drug delivery, and thus for immune modulation and antifungal therapy. The ability to manipulate EV biogenesis or block their release could lead to novel treatments for fungal infections, particularly in the face of rising antifungal resistance. Moreover, understanding the role of EVs in biofilm formation and maintenance could inform the targeting of EV-mediated communication pathways, providing strategies to disrupt these structures, which are often resistant to conventional therapies. Thus, fungal EVs hold great promise for advancing our understanding of fungal pathogenesis and developing novel antifungal therapies, despite ongoing challenges in their isolation and characterization.
